# Active Topological Glass Confined within a Spherical Cavity

**DOI:** 10.1021/acs.macromol.1c02471

**Published:** 2022-01-25

**Authors:** Iurii Chubak, Stanard Mebwe Pachong, Kurt Kremer, Christos N. Likos, Jan Smrek

**Affiliations:** † Faculty of Physics, University of Vienna, Boltzmanngasse 5, A-1090 Vienna, Austria; ‡ Physico-Chimie des Électrolytes et Nanosystèmes Interfaciaux, Sorbonne Université CNRS, F-75005 Paris, France; § Max Planck Institute for Polymer Research, Ackermannweg 10, 55128 Mainz, Germany

## Abstract

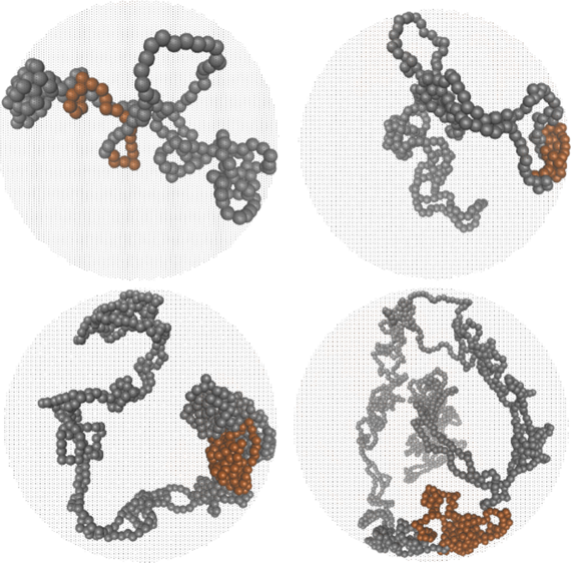

We study active topological glass under spherical confinement, allowing us to exceed the chain lengths simulated previously and determine the critical exponents of the arrested conformations. We find a previously unresolved “tank-treading” dynamic mode of active segments along the ring contour. This mode can enhance active–passive phase separation in the state of active topological glass when both diffusional and conformational relaxation of the rings are significantly suppressed. Within the observational time, we see no systematic trends in the positioning of the separated active domains within the confining sphere. The arrested state exhibits coherent stochastic rotations. We discuss possible connections of the conformational and dynamic features of the system to chromosomes enclosed in the nucleus of a living cell.

## I Introduction

Active topological glass (ATG) is a state of matter composed of polymers with fixed, circular, unknotted topology that vitrifies upon turning a block of monomers active and fluidizes reversibly.^1^ Unlike classical glasses, where the transition is driven by temperature or density, ATG results from physical, tight, threading entanglements, generated and maintained by the activity of polymer segments. The activity acting on the ring segments, modeled here as stronger-than-thermal fluctuations, triggers a directed snakelike motion that overcomes entropically unfavorable states and results in significantly enhanced inter-ring threading.^2^ A topological glass is hypothesized to exist also in equilibrium solutions of sufficiently long ring polymers, where rings naturally thread (pierce through each other’s opening). However, the conjectured critical ring length is currently beyond experimental or computational reach.^3−5^ Although the ATG exhibits accessible critical ring lengths, a formidable challenge in simulating these systems stems from the large system sizes that are necessary to avoid self-threading of significantly elongated partly active rings due to periodic boundary conditions.^1^ To overcome this difficulty, a much smaller system confined to an impenetrable cavity can be simulated. In analogy to classical glasses, where the confinement affects the vitrification mechanism and shifts the glass transition temperature in comparison to the bulk value,^6,7^ it is pertinent to ask the question whether the ATG, the existence of which relies on highly extended configurations that promote intermolecular entanglement, can exist in such a strong confinement at all.

Besides the ATG, the confined melt of uncrossable polymer rings with active segments has an interesting biological connection. The *equilibrium* melt of rings exhibits conformational properties consistent with the large-scale, population-averaged properties of chromatin fiber in the interphase nuclei of higher eukaryotes.^8−10^ In detail, the territorial segregation of distinct chains, the critical exponents ν = 1/3 and γ ≃ 1.1 governing the scaling of the gyration radius *R*(*s*) ∼ *s*^ν^ and the probability of end-contacts *P*(*s*) ∼ *s*^–γ^ of a segment of length *s*, respectively, coincide for the two systems and characterize the so-called fractal (crumpled) globule conformations.^11^ However, similarly to partly active rings, chromatin is out of equilibrium on smaller scales as well. Various processes, such as transcription or loop extrusion, inject energy into the system by the action of respective molecular machines on the chromatin fiber. Fluorescence experiments^12^ and the related analytical theory^13^ suggest that some active events at small scales render fluctuations with thermal spectrum at an effective temperature about twice higher than the ambient one. As an additional gain of our investigation of the confined ATG, we can assess if it can be consistent with the fractal globule model, since both of the latter represent some aspects of the chromatin conformations in space and time.

First, we explore the static and dynamic properties of the long, confined, partly active, nonconcatenated rings in a melt. We find, in agreement with the bulk ATG, arrested conformations in confined systems with a small number of polymer chains. The ability to simulate longer rings than in the bulk allows us to assess in more detail the conformational and scaling properties of the chains in the nonequilibrium glassy state. We discover that intermediate-length ring segments feature conformations consistent with a mean-field statistics of a self-avoiding random walk (ν = 0.588, γ = 1.75). The territorial structure of the fractal globule is distorted and we observe active–passive microphase-separated domains and large-scale correlated motion arising from the glassy phase due to the activity-induced topological constraints. Finally, we observe tank-treading of active segments along the ring contour in the glass that acts to enhance active–passive phase separation when both the chain’s diffusion and the conformational rearrangements are suppressed.

## II Simulation Details

We use the well-established model,^14−16^ in which the excluded volume interaction between any two monomers is described by a repulsive and shifted Lennard-Jones potential
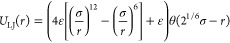
1where θ(*x*) is the Heaviside step function, σ is the bead’s diameter, and ε sets the energy scale. As in ref (10), the same potential was used for the interaction between monomers and the confining sphere of radius *R* that was modeled as a smooth, structureless, purely repulsive barrier. The radius *R* is fixed by the total monomer density ρ = 0.85σ^–3^ for all systems (Table 1). Typically, *R* is about 2.5–2.7 times larger than the equilibrium radius of gyration of the confined chains (Table 3). The polymer bonds were modeled by a finitely extensible nonlinear elastic (FENE) potential
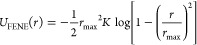
2where *K* = 30.0ε/σ^2^ and *r*_max_ = 1.5σ. These parameters make the chains essentially noncrossable. We also used the angular bending potential
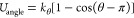
3with the parameter *k*_θ_ = 1.5ε to induce a higher stiffness that corresponds to a lower entanglement length *N*_e_ = 28 ± 1 at the studied monomer density ρ.^15^

**Table 1:** Size and Shape Properties of Partially Active Rings in a Confining Sphere^a^

*N*	*N*_h_	*R*/σ	⟨*R*_g_^2^⟩/σ^2^	⟨*R*_ee_^2^⟩/σ^2^	⟨*R*_ee_^2^⟩/⟨*R*_g_^2^⟩	⟨λ_1_⟩/⟨λ_3_⟩	⟨λ_2_⟩/⟨λ_3_⟩	⟨*R*_g_^2^⟩/*R*^2^	⟨*R*_ee_^2^⟩/*R*^2^
200	25	13.72	62.4(0.7)	164.8(6.2)	2.64	12.0(0.7)	4.3(0.2)	0.33	0.87
400	50	17.29	129.3(0.6)	304.4(6.2)	2.34	6.5(0.4)	3.1(0.7)	0.41	1.01
800	100	21.78	227.7(0.5)	468.6(3.1)	2.05	4.6(0.2)	2.7(0.8)	0.47	0.98
1600	200	27.44	376.1(0.7)	810.8(4.7)	2.15	3.5(0.5)	2.2(0.1)	0.49	1.07

a The mean values as well as their standard errors (indicated in the parentheses) were estimated in the steady states. *N* is the polymer length, *N*_h_ is the number of active (hot) monomers, and *R* is the radius of the sphere.  and  are the mean-square radius of gyration and the mean-square spanning distance between monomers *N*/2 apart, respectively. λ_*i*_ (*i* = 1, 2, 3; λ_1_ ≥ λ_2_ ≥ λ_3_) are the eigenvalues of the gyration tensor.

Our simulations start from well-equilibrated configurations of completely passive ring polymer melts in spherical confinement produced in ref (10). Each system contains *M* = 46 ring polymer chains, each of length *N* (*N* = 200, 400, 800, and 1600, corresponding to chain entanglement number *Z* = *N*/*N*_e_ = 7, 14, 28, and 57), the longest being 4 times longer than the system in ref (1). At time *t* = 0, the activity was introduced by coupling a consecutive segment of length *N*/8 on each ring to a Langevin thermostat at temperature *T*_h_ = 3.0ε, whereas the rest of the ring is still maintained at *T*_c_ = 1.0ε by another Langevin heat bath. We choose this value of *T*_h_ = 3*T*_c_, despite the experimental indications of active fluctuations being only about twice the thermal fluctuations. The reason is that the heat flux between the active and passive constituents establishes effective temperatures that are in between the temperatures set by the thermostat. The effective temperatures (measured by the mean kinetic energy) would be the ones measured in the experiments and have the correct ratio of about 2.^2^ The equation of motion of the systems were integrated using the LAMMPS simulation package^17^ and the velocity Verlet integration scheme with the time step *δt* = 0.005τ and the damping constant γ = 2/3τ^–1^, where τ = σ(*m*/ε)^1/2^.

The Langevin thermostat in spherical confinement can induce stochastic values of angular momentum that affect the real dynamics of the system. This effect can be neutralized by zeroing periodically the total angular momentum during the simulations, as done in equilibrium simulations in ref (10). In the present case, unlike in the equilibrium simulations,^10^ we do not perform this operation due to the nonequilibrium character of the studied system as well as the potential global flows that can arise in active matter states. When compared to dynamic equilibrium quantities across this work, we also used trajectories produced in a similar fashion without zeroing the angular momentum. We note, however, that the difference in dynamic relaxation times in equilibrium simulations with and without zeroing the angular momentum is rather small.

## III Conformational Properties

**Figure 1:**
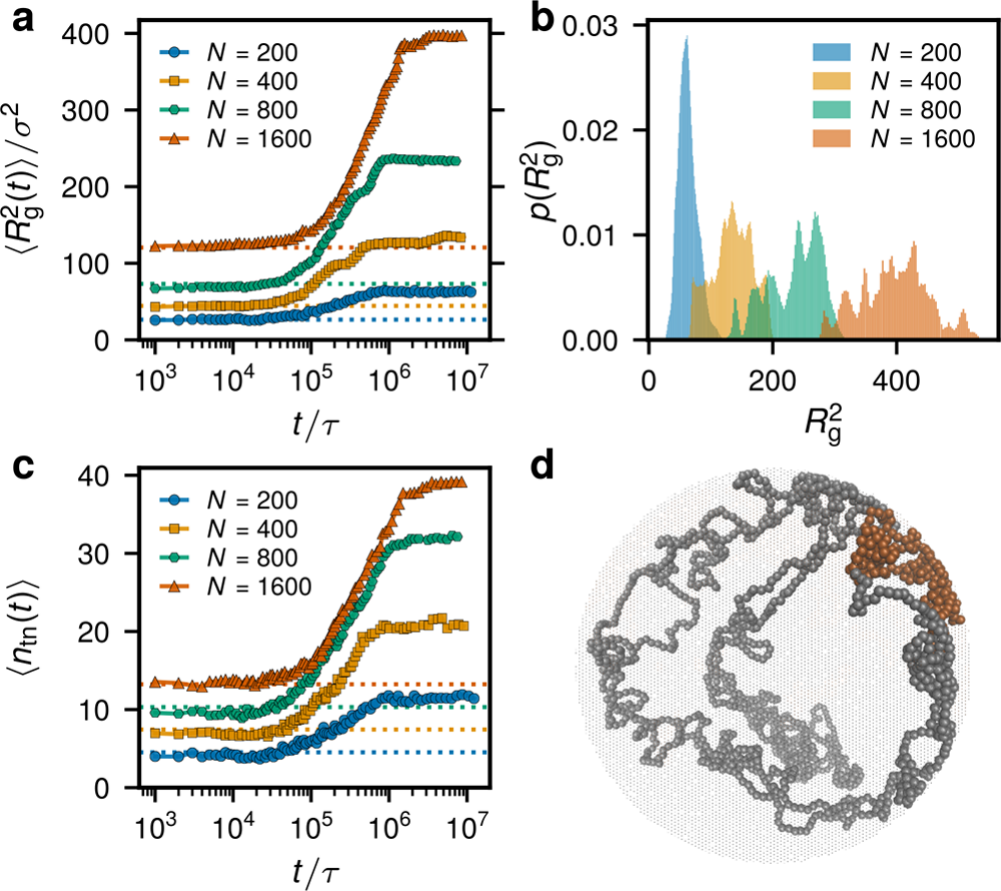
Size and threading properties. (a) Evolution of the ring’s  after the activity onset at *t* = 0 for systems with different *N*. (b) Distribution of  in the steady state. The distributions are time-averaged over the steady state and the one for the *N* = 200 system is averaged also over 10 independent runs. (c) Evolution of the mean number of threaded neighbors. (d) Conformation of a partly active ring with *N* = 1600 at the end of the simulation run. The active and passive monomers are shown with orange and gray, respectively. In parts a and c, the dashed lines of the respective colors indicate the values in equivalent equilibrium ring melts.^10^

When the activity is switched on, after about 10^5^τ, the chains start to expand from their equilibrium sizes until they reach a steady state after (2–3) × 10^6^τ. The time of the onset of the chain stretching does not significantly depend on *N* because it is related to local threading constraints. The steady state is characterized by a significantly enhanced mean-square radius of gyration  (see the snapshot of a chain in Figure 1d, the time evolution of  in Figure 1a, and Table 1 for a shape parameter comparison). The steady states exhibit a rugged distribution of  (Figure 1b), despite averaging over about 10^7^τ, time that is more than 1 order of magnitude above the equilibrium diffusion times for *N* ≤ 800. This shows that the individual chains are not able to change their conformations significantly, being essentially frozen in the same state, and points to a nonergodic behavior. When averaged over 10 independent runs, a smoother distribution is recovered, as shown for *N* = 200 in Figure 1b.

The stretching due to the snakelike motion after the activity onset is caused by the strong dynamic asymmetry between the active and the passive segments, apparently triggered by nonequilibrium phase separation.^1,2,18^ The dynamics of the mutual ring threading coincides with the stretching dynamics and exhibits markedly enhanced numbers of threaded neighbors *n*_tn_ by a single ring in the steady state in comparison to equilibrium (Figure 1c), as we showed by analyzing piercings of rings through other rings’ minimal surfaces.^1,10,19−21^ This method has been used successfully to analyze threading constraints for systems containing ring polymers in^10,19,21^ and out of equilibrium.^1,2,20^ The essence of the method is an unambiguous definition of the threading as the intersection of a rings contour with a minimal disklike surface spanned on another ring. For the details on practical implementation of the algorithm, we refer the reader to refs (1) and (20). Interestingly, the number of threaded neighbors is the same as for the active topological glass in the bulk,^1^ despite the different ring shape (compare Tables 1 and 2). For the longest rings, each ring practically threads all the other rings in the system.

**Table 2:** Size and Shape Properties of the Partially Active Rings in Bulk^a^

*N*	*N*_h_	⟨*R*_g_^2^⟩/σ^2^	⟨*R*_e_^2^⟩/σ^2^	⟨*R*_ee_^2^⟩/⟨*R*_g_^2^⟩	⟨λ_1_⟩/⟨λ_3_⟩	⟨λ_2_⟩/⟨λ_3_⟩
100	13	18.1(0.1)	54.9(0.1)	3.0	7.3(0.1)	2.34(0.01)
200	25	65.2(0.3)	203.5(3.8)	3.1	12.4(0.1)	2.81(0.01)
400	50	182.1(0.7)	566.1(2.1)	3.1	14.2(0.2)	3.03(0.02)

a *N* is the polymer length and *N*_h_ is the number of active (hot) monomers.  is the mean-square radius of gyration,  is the mean-square distance between two monomers separated by the contour length *N*/2, and λ_*i*_ (*i* = 1, 2, 3) are the eigenvalues of the gyration tensor ordered such that λ_1_ ≥ λ_2_ ≥ λ_3_. The value in parentheses indicates the standard error. Adapted from ref (2).

**Table 3:** Size and Shape Properties of the Equilibrium Confined Rings^a^

*N*	*R*/σ	⟨*R*_g_^2^⟩/σ^2^	⟨*R*_e_^2^⟩/σ^2^	⟨*R*_ee_^2^⟩/⟨*R*_g_^2^⟩	⟨λ_1_⟩/⟨λ_3_⟩	⟨λ_2_⟩/⟨λ_3_⟩
200	13.72	26.4(0.2)	73.4(0.6)	2.8	5.64(0.04)	2.25(0.01)
400	17.29	44.4(0.7)	120.7(2.5)	2.7	5.24(0.08)	2.14(0.02)
800	21.78	73.1(1.1)	195.4(3.8)	2.7	4.93(0.10)	2.06(0.01)
1600	27.44	120.5(2.8)	320.2(10.4)	2.7	4.89(0.12)	2.03(0.02)

a *N* is the polymer length and *R* is the radius of the confining sphere,  is the mean-square radius of gyration,  is the mean-square distance between two monomers separated by the contour length *N*/2, and λ_*i*_ (*i* = 1, 2, 3) are the eigenvalues of the gyration tensor ordered such that λ_1_ ≥ λ_2_ ≥ λ_3_. The value in parentheses indicates the standard error. Adapted from ref (10).

**Figure 2:**
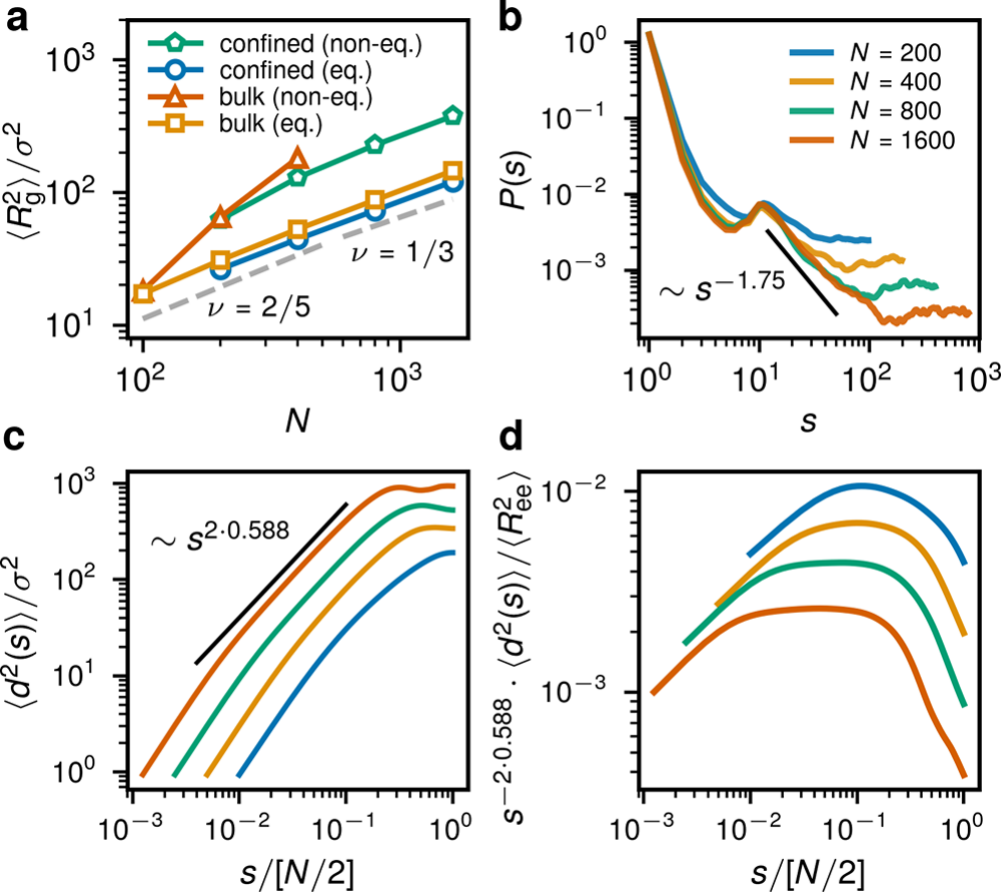
Conformational properties. (a) Comparison of the scaling of the radius of gyration with the ring length *N* for different systems. The results for the confined active rings are from the present work, the confined equilibrium rings are from ref (10), the bulk equilibrium are from ref (15), and the bulk active rings are from ref (1). The equilibrium scaling with the exponent ν = ^1^/_3_ is shown as well as the crossover with the “effective exponent” ^2^/_5_. (b) The contact probability *P*(*s*) for different *N*. At intermediate distances {*s*/(*N*/2) ∈ [10^–2^; 10^–1^]} and for long rings we recover the exponent γ close to 1.75, consistent with the self-avoiding random walk configurations. (c) The mean-square internal distance ⟨*d*^2^(*s*)⟩ for different *N*. (d) The mean-square internal distance ⟨*d*^2^(*s*)⟩ multiplied by *s*^–2×0.588^ and normalized by the mean-square end-to-end distance  (Table 1). The broadening plateau for the rings of *N* ≥ 400 shows the asymptotic self-avoiding regime. The legend in parts c and d is the same as that in part b.

In comparison to the bulk ATG,^1^ the confined rings here are significantly less expanded in terms of *R*_g_ and the ratio of the two biggest eigenvalues of the gyration tensor (Figure 2a and compare Tables 1 and 2 for *N* = 400). Note that the seeming compact scaling of the active confined rings, as seen in Figure 2a, is not due to their internal structure (Figure 2b–d), but just because the confining radius *R* scales with *N*^1/3^ as the systems of different *N* were simulated with the same number of chains and the same density.

**Figure 3:**
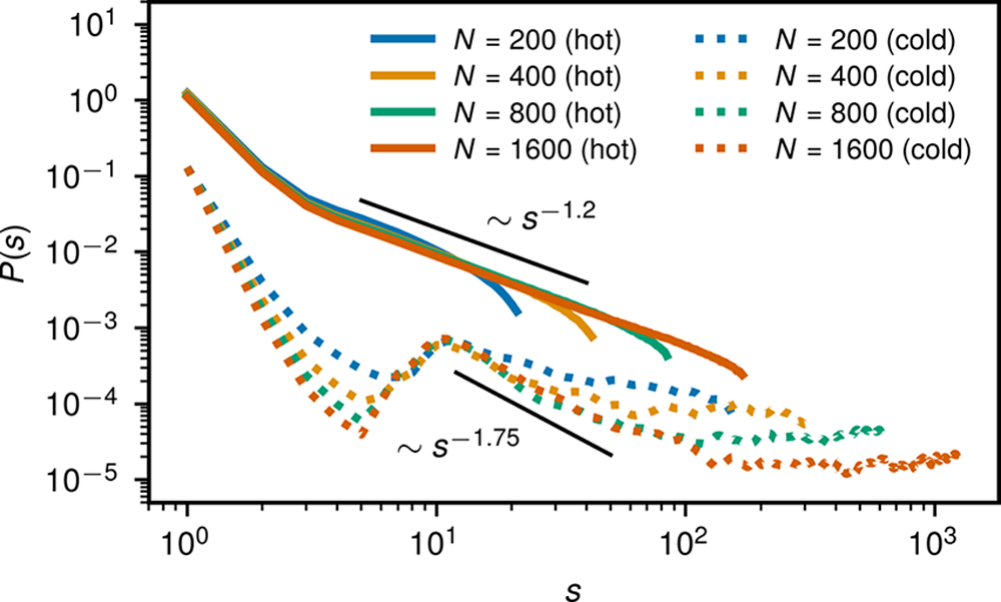
Segment-resolved contact probability. *P*(*s*) was computed separately for hot (solid lines) and cold (dotted lines) segments. The dotted lines have been shifted vertically by 1 decade for clarity.

As compared to the equilibrium case^10^ and seen in Figure 2b, the rings are highly stretched and exhibit a scaling of the contact probability *P*(*s*) ∼ *s*^–γ^ with the exponent γ close to 1.75 at intermediate distances {*s*/(*N*/2) ∈ [10^–2^; 10^–1^]} and a plateau at largest distances, signifying the loss of correlation due to reflections of rings from the walls. *P*(*s*) was computed as the probability of finding the end points of a segment *s* at distance below 2^1/6^σ and averaged over the segment’s position within a ring and over different rings in the steady state. Due to the phase separation of the hot and cold segments and the doubly folded chain structure, *P*(*s*) features a nonmonotonic character. Resolving the contact probability for the active and the passive case separately, we found exponents 1.2–1.33 for the active part and 1.75 for the passive one (see Figure 3). While the former is consistent with a crumpled globule with a relatively smooth interface (due to microphase separation),^9^ the latter has self-avoiding walklike features. The exponent γ = 1.75 at intermediate *s* is consistent with a mean-field estimate of γ = *νd* ≃ 1.76, with ν = 0.588 and *d* = 3 being the dimensions of space.^9^ This estimate neglects correlation effects in isolated self-avoiding walks, which when taken into account yield an asymptotic scaling exponent γ ≃ 2.18.^22^ We surmise that the difference is not coincidental and can be caused by two effects: (i) the length scale at which we observe the scaling is not long enough to display the true asymptotic scaling or (ii) partial screening of the excluded volume caused by the presence of other chains in the melt can alter the exponent. We leave the resolution of these interesting facts for future studies.

The observed ring conformations are mostly doubly folded (e.g., see Figure 1d) and the change in the shape parameters (Table 1) is due to “reflections” from the walls. This can be seen in the mean-square internal distance ⟨*d*^2^(*s*)⟩ of the longest rings (Figure 2c) being a nonmonotonic function of the contour length. ⟨*d*^2^(*s*)⟩ was computed as the mean square end-to-end vector of a segment of length *s* averaged over its position within a ring and over different rings in the steady state. As shown in Figure 2d, at intermediate distances {*s*/(*N*/2) ∈ [10^–2^; 10^–1^]} we recover the self-avoiding walk scaling exponent 0.588 for the longer (*N* ≥ 400) rings, in agreement with the results for *P*(*s*) in Figure 2b. In summary, the stretched conformations and the different profile of the contact probability signifies the loss of the original crumpled globule characteristics of equilibrium rings.^9^

## IV Active–Passive Microphase Separation

**Figure 4:**
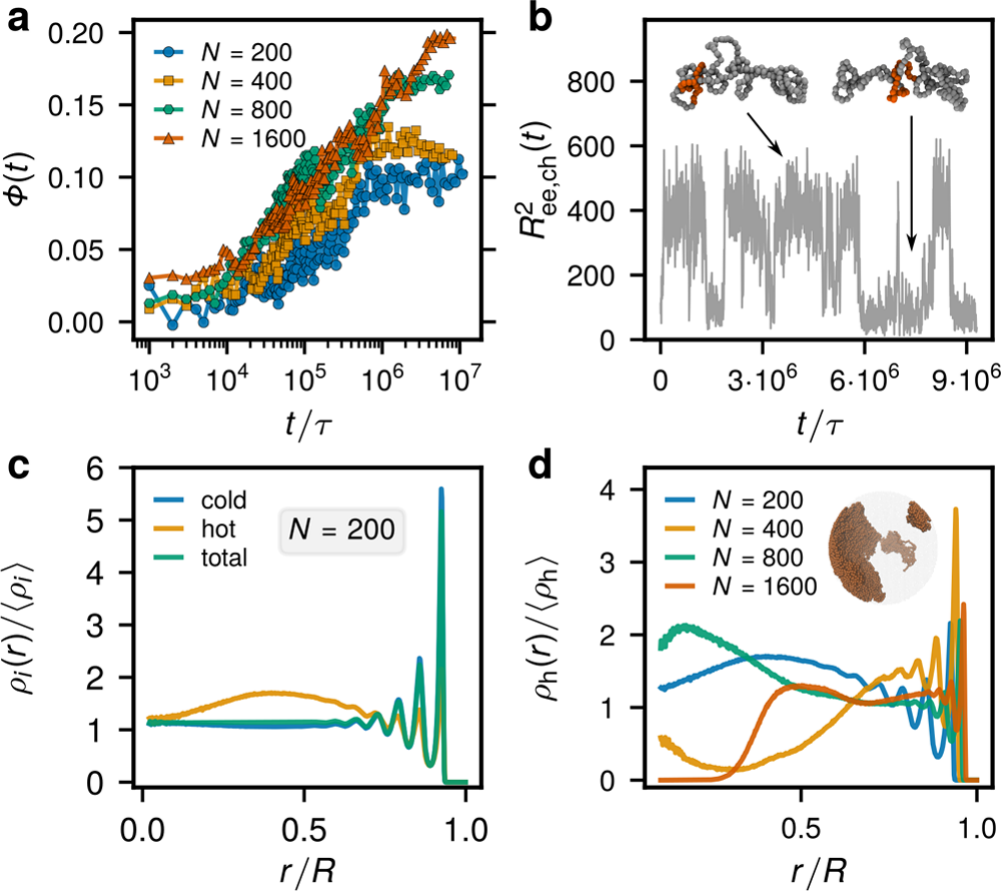
Phase separation. (a) Time evolution of the phase separation order parameter Φ(*t*). (b) Time dependence of  for one of the rings illustrating tank-treading motion. (c) Radial distribution of cold (blue), hot (yellow), and all (green) monomers within the enclosing sphere for the system with *N* = 200 (averaged over 10 independent runs). (d) Radial distributions of hot monomers. Inset: phase-separated regions of hot monomers (cold not shown for clarity) for the system with *N* = 1600.

The chosen model parameters trigger active–passive microphase separation in all the systems.^2,18,23^ Note that this is not an effect of different stiffnesses of the active/passive blocks but a genuine nonequilibrium effect (as evidenced by a comparison to simulations of mixtures of chains with different stiffnesses^18,24^ and also by the agreement of the simulations^2,18^ with the analytical result for the dependence of the critical activity ratio on polymer length^25^). We track the degree of phase separation by the order parameter Φ(*t*) = *x*(*t*)/*x*(0) – 1, where *x*(*t*) is the number fraction of interchain like-particles (particles of the same type, but belonging to different chains) in a *r*_c_ = 2^1/6^σ neighborhood of a given monomer at a given time *t*, averaged over monomers (Figure 4a). The initial increase of the order parameter precedes the ring stretching and threading dynamics, supporting the conjecture in ref (2) that the separation tendency is a precursor of the formation of the glass. The phase separation is dynamic in nature, showing intervals of a single mostly hot region, but also subsequent dissociation into several hot blobs (Supporting Video 1) reminiscent of the dynamics of activity-driven colloidal crystals.^26^ When the shape properties arrive at a steady state, there are several hot blobs (see the inset of Figure 4d) and we still observe them occasionally exchanging hot particles. We discover that these are the consequence of a rare tank-treading motion of some of the rings, by which the hot segment joins the hot phase without changing the overall shape of the ring and the system as a whole. The squared (“end-to-end”) distance  between an active and a passive monomer separated by a segment of length *N*/2 in Figure 4b shows that the tank-treading, a tangential motion of the hot segment along the ring’s contour, is indeed observed. Strikingly, in contrast to classical polymer glasses, where each monomer is caged by its neighbors, the tank-treading mechanism in ATG can enhance active–passive phase separation even when both conformational and diffusional relaxation of polymers are not possible.

Two studies^27,28^ report a preferential positioning of hot monomers within the confining volume, central or peripheral depending on the overall density.^27,28^ In contrast to our work, these works do not report activity-enhanced topological constraints and the resulting arrested dynamics (possibly as the consequence of using a single linear chain^27^ or cross-linked chains with short active segments^28^). To assess the positioning in our *arrested* steady states we computed the radial density distribution of the hot monomers averaged over 10 different runs for *N* = 200. It displays confinement-induced layering at the wall as in equilibrium,^10^ and another broad maximum around *R*/2 (Figure 4c). However, the analysis of single runs for *N* = 200 and for other *N* values shows that the positioning of hot monomers is history-dependent, arrested by the topological constraints, and allows for both internal or peripheral locations (Figure 4d) in contrast to a preference for central locations of active monomers in a different polymer model in ref (28).

## V Dynamics and Relaxation

**Figure 5:**
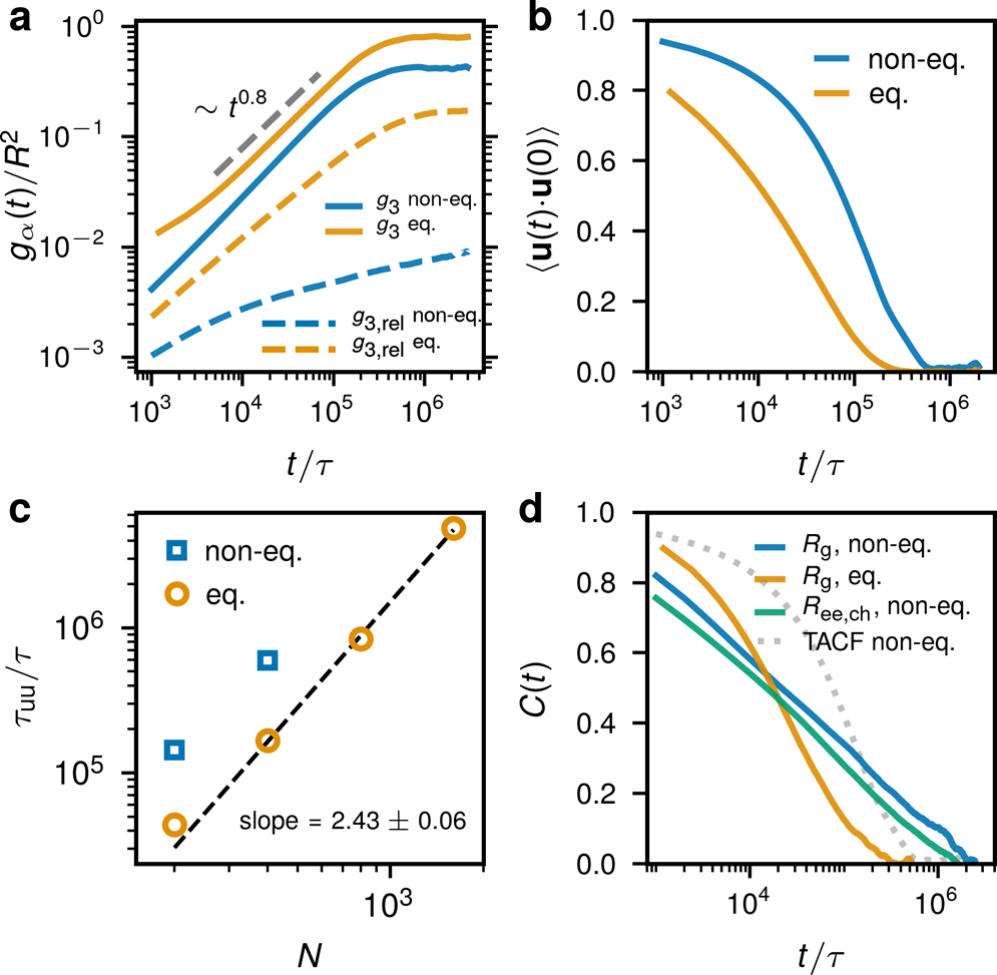
Dynamics and relaxation. Comparison of the nonequilibrium system (blue) with the equilibrium one (yellow). (a) Mean-square displacements of the ring’s center of mass *g*_3_ normalized by the squared sphere’s radius *R*^2^ of *N* = 200 (solid lines). The relative mean-square displacement *g*_3,rel_(*t*) (dashed). (b) Terminal autocorrelation function for the system with *N* = 200. (c) The relaxation time of the terminal autocorrelation function, τ_uu_ = *∫*d*t*⟨**u**(*t*)·**u**(0)⟩, for equilibrium (yellow circles) and nonequilibrium active (blue squares) confined rings of different length *N*. The data for equilibrium confined rings was taken from ref (10). (d) Normalized autocorrelation function for  for *N* = 200. For comparison, the autocorrelations of the squared “end-to-end” (i.e., between an active and a passive monomer separated by segment length *N*/2) distance  (solid green) and the TACF (dashed gray) are shown. In all cases, we subtract the mean value squared and normalize the autocorrelation functions to unity at time zero.

In Figure 5 we report dynamical and relaxation properties of rings in the system with *N* = 200 (averaged over 10 independent runs). We focus on the steady-state dynamics by computing the mean-square displacements of the ring’s center of mass, *g*_3_(*t*):
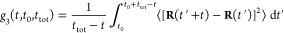
4where *t*_0_ is the initial time point chosen as the onset of the steady state (3 × 10^6^τ in the case of active rings and 0 for equilibrium systems), *t*_tot_ is the total simulation time, **R** is the position of the ring’s center of mass with respect to the global center of mass, and the angled brackets ⟨...⟩ mean averaging over different rings. As shown in Figure 5a, the late-stage (steady-state) dynamics of the ring’s center of mass is much slower than in the equilibrium case,^10^ with negligible relative displacements between the rings *g*_3,rel_(*t*), with the latter defined as follows:
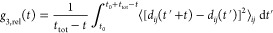
5where *t*_0_ and *t*_tot_ are as above, *d*_*ij*_ is the relative *distance* between the centers of mass of rings *i* and *j* and the ⟨...⟩_*ij*_ is the average over all possible ring pairs in the system. Importantly, *g*_3,rel_(*t*) is invariant under overall constant global rotations and shows that the relative ring’s motion essentially stalls. The systems with longer rings display the same behavior. In confined systems, *g*_3_(*t*) saturates at a constant value; for the rings with *N* = 200, we find that *g*_3_(*t*→*∞*) ≈ 0.4*R*^2^, which is about 2 times smaller than in the equivalent equilibrium case (Figure 5a). This arises mostly from extremely elongated and practically frozen rings conformations, due to which the exploration of the available volume is significantly suppressed (Supporting Video 2).

In Figure 5b, we characterize the ring structural relaxation by considering the terminal autocorrelation function (TACF) ⟨**u**(*t*)·**u**(0)⟩, where **u**(*t*) is the unit vector connecting two monomers separated by contour distance *N*/2, and the average is taken over all such monomer configurations within a ring and time.^16,29^ As seen in Figure 5b,c, the decorrelation time of the TACF τ_uu_ = *∫*d*t*⟨**u**(*t*)·**u**(0)⟩ is about 3 times longer than in the counterpart equilibrium cases. In the steady state, the rings are found in a heavily threaded arrangement with their configurations being essentially frozen, as evidenced by the static properties in Figure 1. Since in the steady state the relative ring displacements are marginal (Figure 5a), ⟨**u**(*t*)·**u**(0)⟩ can decorrelate either through internal conformational ring relaxation or collective system rotations.

In what follows, we show that the main pathway that contributes to the decorrelation of the TACF are correlated, stochastic rotations of the whole system. The other possible decorrelation mechanism is the internal ring rearrangements, caused by the local explorations of the hot segments or tank-treading motion. To show that these do not dominate, we show in Figure 5d that the normalized autocorrelation function for the ring’s , *C*(*t*), fully decorrelates at a later time (≈2 × 10^6^τ) compared to the TACF (≈6 × 10^5^τ) and features a 3 decades long logarithmic decay. This contrasts with the equilibrium behavior, where both structural quantities  and ⟨**u**(*t*)·**u**(0)⟩ decorrelate at about the same time (yellow curves in Figure 5b,d). Although the size of the rings essentially stays the same during the TACF relaxation, there remains the possibility of tank-treading motion that can significantly impact the TACF decorrelation but keep the overall size given by *R*_g_ fixed. We show that the tank-treading does not significantly impact the terminal relaxation by computing the autocorrelation function for the squared end-to-end distance  (Figure 5d). Although it decorrelates slightly faster than the one for , its relaxation time is still larger than that of the TACF. Therefore, collective, stochastic rotations provide the dominant contribution to the TACF decorrelation, whereas its relaxation time scale can be used as an estimate for the rotational diffusion time (the presence of such global, correlated rotations is visible in both Supporting Videos).

**Figure 6:**
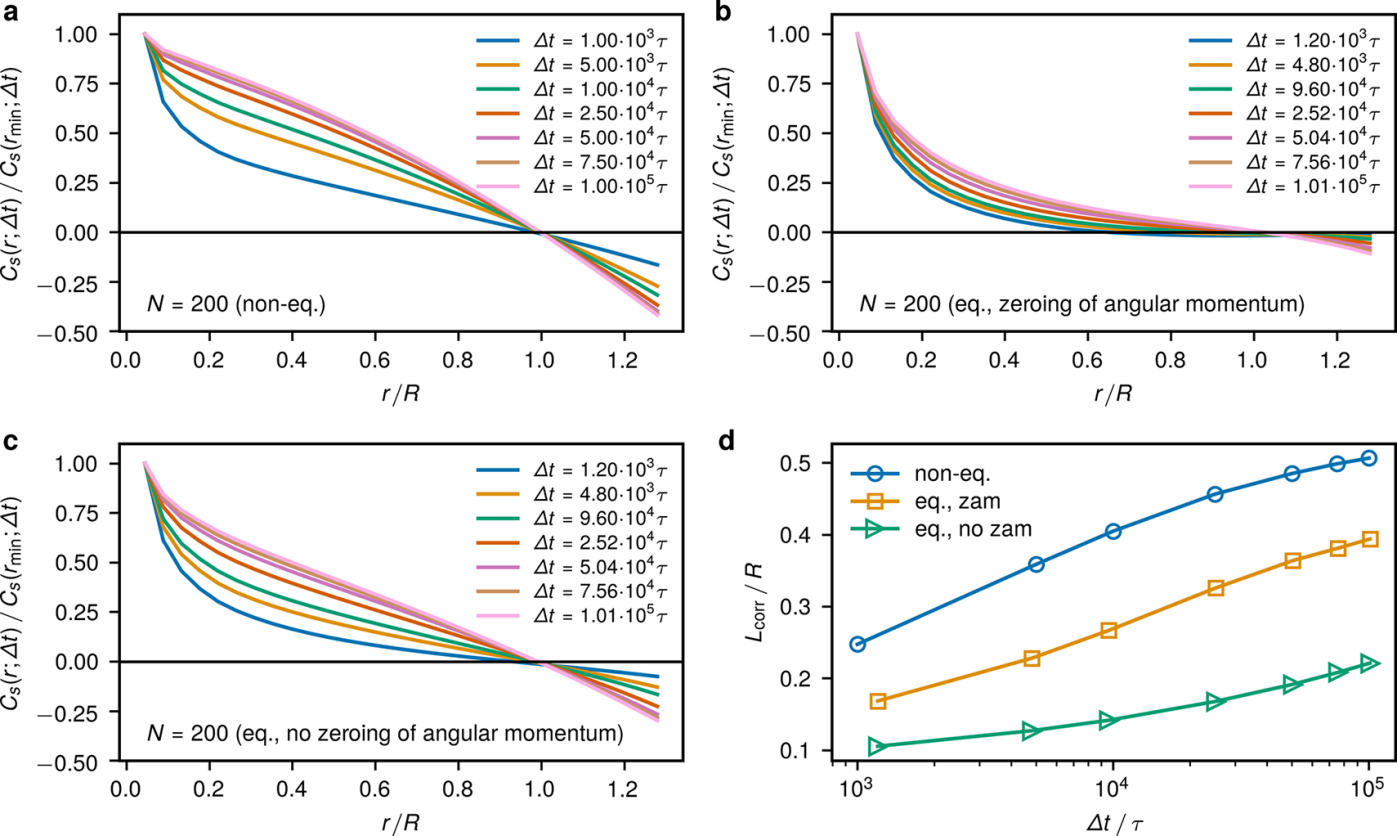
Spatiotemporal displacement correlation. The correlation function *C*_s_(*r*;Δ*t*) (eq 6) for *N* = 200 in the case of (a) active confined rings, (b) equilibrium rings with zeroing of the system’s angular momentum, and (c) equilibrium rings without zeroing of the angular momentum. The equilibrium systems are taken from ref (10). For a given Δ*t*, the shown spatiotemporal correlation functions are normalized by their value at the *r*-bin centered at *r*_min_ = 0.9σ. (d) The correlation length  scaled with the confinement radius *R* as a function of the lag time Δ*t* for the latter three cases.

Global rotations lead to correlated particle displacements, as detailed by computing the spatiotemporal correlation function similarly to^12,30^
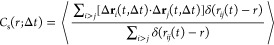
6where Δ**r**_*i*_(*t*,Δ*t*) is the displacement of the *i*th monomer in lag time Δ*t* starting from time *t*, Δ**r**_*i*_(*t*,Δ*t*) = **r**_*i*_(*t*+Δ*t*) – **r**_*i*_(*t*), and *r*_*ij*_(*t*) = |**r**_*i*_(*t*) – **r**_*j*_(*t*)|. Numerically, *C*_s_(*r*;Δ*t*) was computed with a spatial resolution of 0.6σ. The angular brackets represent averaging over time, in the active case only over the steady state. In the active system, the correlation decays significantly slower in comparison to equilibrium and there is a strong anticorrelation at longer lag times at the opposing positions in the spherical confinement (*r* > *R*), as seen in Figure 6a–c. In part, this is a consequence of the Langevin dynamics that induces stochastic angular momentum also in equilibrium (Figure 6c). However, the anticorrelation is much more pronounced in the active topological glass state and almost nonexistent in equilibrium with zeroed angular momentum (Figure 6b). Finally, the pronounced correlation of particle displacements in the active case results in the increased effective correlation length  compared to the passive system (Figure 6d). Note that in the definition of *L*_corr_ the correlation function (eq 6) was normalized by its value at the first spatial bin considered with *r* ∈ [0.6σ, 1.2σ] centered at *r*_min_ = 0.9σ.

## VI Discussion

The ATG represents a novel class of polymer glass. The activity-enhanced topological constraints inhibit the center of mass diffusion of the rings, as well as their conformational relaxation, but allow for displacements of the monomers along the contour of the rings. As we have shown here, this tank-treading diffusion can alter the underlying microphase separation but does not seem to affect the overall stability of the ATG. Given the ATG formation mechanism, by which the active segment pulls tight the topological constraints,^1,2^ one could naïvely expect the ATG to fluidize when the active segment tank-treads significantly along the contour. We hypothesize that the stability is maintained by the high number of “redundant” constraints each chain has with other chains, which then do not allow for a simple unthreading. For the system to liquidify again, many unthreading events would have to occur sequentially in the opposite order of their formation. The microphase separation tendencies colocalize active segments, making it highly unlikely for these to tank-tread independently and to alter the stability. Yet, to judge this picture more work has to be done. In particular, we would need to understand better the geometrical and topological nature and the spatial distribution of the constraints and their role in the ATG stability. Despite the fact that a number of threading detection methods already exist,^31−33^ none so far is tuned to detect all the latter aspects. Such a tool would help to clarify also the conjectured existence of topological glass in equilibrium, where it should arise in the limit of long rings, as suggested by simulations^3−5^ and analytical works.^34,35^ A theory for the ATG is highly desirable, but an extension of the equilibrium works is not straightforward because they rely on the compact equilibrium ring conformations. As we detail in this work, the conformations of rings in ATG are not compact but highly stretched, self-avoiding, and walklike (therefore the topological constraints are also likely very distinct from equilibrium). Another related intriguing result that merits future investigations is the different scaling of the contact probability of true self-avoiding walk (γ ≃ 2.18) and the one we found in ATG conformations (γ ≃ 1.75) that is consistent with the simple estimate *νd*.

Let us now turn to a discussion of a possible connection of the large correlated motions (rotational diffusion) in ATG and coherent motion of chromatin on the micron scale, observed in refs (12) and (36), interpreted also as rotations of the nucleus interior.^37^ Various mechanisms involving activity have been proposed to cause large-scale correlated motions.^28,30,38−42^ The works^30,39,41^ focus on the spatiotemporal correlations and found large-scale correlated motions, but of different origins. In ref (30), the correlated domains coincide with the microphase separated domains due to preferential intradomain interaction, and the (thermal-like) activity opposes the coherence, similarly to ref (40). The correlated motion in ref (39) comes from the coupling of the hydrodynamic flow due to contractile motor activity and the nematic ordering of the chromatin fiber (not yet observed). The contractile motor activity in ref (41) generates the correlated motion as a result of a high number of cross-links (interactions) between chromatin fibers and chromatin and a deformable nuclear envelope. Moreover, the nature of the correlated motion can depend on the form of the activity (e.g., thermal-like or force dipoles), and distinct active correlations can arise even along the contour of a single phantom (no nonbonded interactions) chain.^43^ Last but not least, apart from the role of activity in correlated motions, other, passive mechanisms are possible.^44,45^ The latter work also highlights glassy features of the chromatin dynamics, such as dynamic heterogeneity.

Clearly, our simplified system is not directly applicable to chromatin (e.g., the large-scale conformational data are different). Nevertheless, the confined ATG shows yet another mechanism how correlated motion can arise: the activity enhances the entanglement between neighboring domains that forces them to move in a correlated fashion. In contrast to the chromatin models in refs (30) and (41), where the large-scale dynamical coherence arises from explicit interaction potentials or cross-links, here we show that the activity-induced entanglement can mediate the correlated motion as well. As we do not model chromatin in detail, we can compare just qualitatively the dynamical features of the correlated motion: in ATG we observe correlation length to be larger with activity than in the passive case (Figure 6d), which is consistent with refs (12), (36), and (41) but contrasts with ref (30). However, the correlation length is monotonically increasing and saturating with time, which is consistent with some cases in ref (36), but nonmonotonic correlation length has been observed in other cases at longer time lags.^12,30,36,41^

More work is necessary to find out if other types of topological glass (dynamic correlations arising from entanglements) can be consistent with conformational data. At length scales below 1 Mbp the chromatin has nontrivial topology (due to cohesin-mediated loops)^46^ and therefore might be subject to mutual threading topological constraints. Simulations with finer resolution and diverse distribution of the active segments would be necessary to give a satisfactory answer to the connection. A notable work in this context^30^ uses active sites distributed along the polymer according to the epigenetic information on a given chromosome that is modeled as an uncrossable chain with initially fractal–globule large-scale conformational properties. However, the work does not report entanglements or conformational changes of the active segments. Despite some active segments being long enough (20–80 beads) for activity-driven microphase separation,^18^ the relatively lower density, in comparison to ours, and a differential interaction of the active and the inactive chromatin types could suppress or obscure the activity-driven conformational changes we see in ATG. Also for these reasons a more detailed understanding of topological constraints in dense mixtures of active–passive copolymers with nontrivial topology is of major importance. A step in that direction would be to construct a phase diagram (in terms of activity contrast, density, and block lengths) of the ATG.

From the materials research perspective, active copolymers represent a very promising direction in macromolecular science. The interplay of topology, activity, and (active) microphase separation promises a concurrent control of the entanglement, dynamics, and the morphology of the system. The ATG is a prototypical example of this broader class of prospective materials. Our work shows that the ATG can be efficiently explored at a significantly reduced computational costs in confinement. We characterized the chain static properties and discovered the tank-treading relaxation mechanism that, however, does not seem to affect the glass stability but only the phase-separation properties. A more detailed understanding of the topological constraints maintaining the ATG stability should be gained in future to experimentally synthesize ATG and fully characterize this novel dynamical transition.

## Figures and Tables

**Figure 7:**
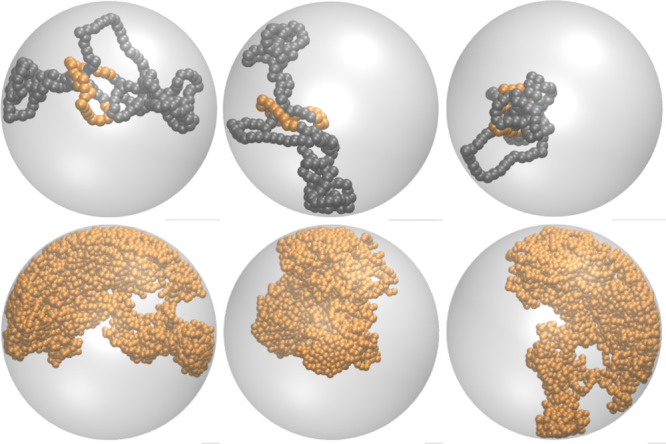
Steady-state snapshots. Top row: A single chain of *N* = 200 viewed from three different angles. The active and passive monomers are shown with orange and gray, respectively. Bottom row: The system with *N* = 800, where only the hot monomers are shown for clarity of the spatial distribution.

## Appendix A

In this appendix, we summarize the size and shape parameters of partially active rings in bulk (Table 2) and of equilibrium confined rings (Table 3) in the melt that are mentioned throughout the paper.

Additionally, in Figure 7 we present more steady-state snapshots of the systems.

## Supporting Information Available

The phase separation between active and passive blocks after the activity onset in the confined system with *N* = 1600. Only active monomers (orange) are shown for clarity (Supporting Video 1) (MPG)
The dynamics of a single confined partly active ring of length *N* = 1600 after the activity onset. The active monomers are orange and the passive ones gray (Supporting Video 2) (MPG)

## Supplementary Material

ma1c02471_si_001.mpg

ma1c02471_si_002.mpg

## Notes

The relevant data sets generated and/or analyzed in the current study are available from the corresponding authors on reasonable request.

## References

[ref1] Smrek J.; Chubak I.; Likos C. N.; Kremer K. Active topological glass. Nat. Commun. 2020, 11, 26 10.1038/s41467-019-13696-z. 31911582 PMC6946665

[ref2] Chubak I.; Likos C. N.; Kremer K.; Smrek J. Emergence of active topological glass through directed chain dynamics and nonequilibrium phase segregation. Phys. Rev. Research 2020, 2, 043249 10.1103/PhysRevResearch.2.043249.

[ref3] Michieletto D.; Turner M. S. A topologically driven glass in ring polymers. Proc. Natl. Acad. Sci. U.S.A 2016, 113, 5195 10.1073/pnas.1520665113. 27118847 PMC4868430

[ref4] Michieletto D.; Nahali N.; Rosa A. Glassiness and heterogeneous dynamics in dense solutions of ring polymers. Phys. Rev. Lett. 2017, 119, 197801 10.1103/PhysRevLett.119.197801. 29219489

[ref5] Lo W.-C.; Turner M. S. The topological glass in ring polymers. EPL 2013, 102, 58005 10.1209/0295-5075/102/58005.

[ref6] Jackson C. L.; McKenna G. B. The glass transition of organic liquids confined to small pores. J. Non-Cryst. Solids 1991, 131–133, 221 10.1016/0022-3093(91)90305-P.

[ref7] Pissis P.; Daoukaki-Diamanti D.; Apekis L.; Christodoulides C. The glass transition in confined liquids. J. Phys.: Condens. Matter 1994, 6, L325 10.1088/0953-8984/6/21/008.

[ref8] Rosa A.; Everaers R. Structure and dynamics of interphase chromosomes. PLOS Comput. Biol. 2008, 4, e1000153 10.1371/journal.pcbi.1000153. 18725929 PMC2515109

[ref9] Halverson J.; Smrek J.; Kremer K.; Grosberg A. From a melt of rings to chromosome territories: The role of topological constraints in genome folding. Rep. Prog. Phys. 2014, 77, 022601 10.1088/0034-4885/77/2/022601. 24472896

[ref10] Pachong S. M.; Chubak I.; Kremer K.; Smrek J. Melts of nonconcatenated rings in spherical confinement. J. Chem. Phys. 2020, 153, 064903 10.1063/5.0013929. 35287461

[ref11] Grosberg A. Y.; Nechaev S.; Shakhnovich E. The role of topological constraints in the kinetics of collapse of macromolecules. J. Phys. (Paris) 1988, 49, 2095 10.1051/jphys:0198800490120209500.

[ref12] Zidovska A.; Weitz D. A.; Mitchison T. J. Micron-scale coherence in interphase chromatin dynamics. Proc. Natl. Acad. Sci. U.S.A 2013, 110, 15555 10.1073/pnas.1220313110. 24019504 PMC3785772

[ref13] Bruinsma R.; Grosberg A. Y.; Rabin Y.; Zidovska A. Chromatin hydrodynamics. Biophys. J. 2014, 106, 1871 10.1016/j.bpj.2014.03.038. 24806919 PMC4017295

[ref14] Kremer K.; Grest G. S. Dynamics of entangled linear polymer melts: A molecular-dynamics simulation. J. Chem. Phys. 1990, 92, 5057 10.1063/1.458541.

[ref15] Halverson J. D.; Lee W. B.; Grest G. S.; Grosberg A. Y.; Kremer K. Molecular dynamics simulation study of nonconcatenated ring polymers in a melt. I. Statics. J. Chem. Phys. 2011, 134, 204904 10.1063/1.3587137. 21639474

[ref16] Halverson J. D.; Lee W. B.; Grest G. S.; Grosberg A. Y.; Kremer K. Molecular dynamics simulation study of nonconcatenated ring polymers in a melt. II. Dynamics. J. Chem. Phys. 2011, 134, 204905 10.1063/1.3587138. 21639475

[ref17] Plimpton S. Fast parallel algorithms for short-range molecular dynamics. J. Comput. Phys. 1995, 117, 1–19. 10.1006/jcph.1995.1039.

[ref18] Smrek J.; Kremer K. Small activity differences drive phase separation in active–passive polymer mixtures. Phys. Rev. Lett. 2017, 118, 098002 10.1103/PhysRevLett.118.098002. 28306285

[ref19] Smrek J.; Grosberg A. Y. Minimal surfaces on unconcatenated polymer rings in melt. ACS Macro Lett. 2016, 5, 750 10.1021/acsmacrolett.6b00289. 35614671

[ref20] Smrek J.; Kremer K.; Rosa A. Threading of unconcatenated ring polymers at high concentrations: Double-folded vs time-equilibrated structures. ACS Macro Lett. 2019, 8, 155 10.1021/acsmacrolett.8b00828. 30800531 PMC6383510

[ref21] Rosa A.; Smrek J.; Turner M. S.; Michieletto D. Threading-induced dynamical transition in tadpole-shaped polymers. ACS Macro Lett. 2020, 9, 743 10.1021/acsmacrolett.0c00197. 33828901 PMC8016395

[ref22] des Cloizeaux J. Short range correlation between elements of a long polymer in a good solvent. J. Phys. (Paris) 1980, 41, 223 10.1051/jphys:01980004103022300.

[ref23] Smrek J.; Kremer K. Interfacial properties of active–passive polymer mixtures. Entropy 2018, 20, 520 10.3390/e20070520. PMC7513047 33265609

[ref24] Kozuch D. J.; Zhang W.; Milner S. T. Predicting the flory-huggins χ parameter for polymers with stiffness mismatch from molecular dynamics simulations. Polymers 2016, 8, 241 10.3390/polym8060241. PMC6432250 30979334

[ref25] Ilker E.; Joanny J.-F. Phase separation and nucleation in mixtures of particles with different temperatures. Phys. Rev. Res. 2020, 2, 023200 10.1103/PhysRevResearch.2.023200.

[ref26] Palacci J.; Sacanna S.; Steinberg A. P.; Pine D. J.; Chaikin P. M. Living crystals of light-activated colloidal surfers. Science 2013, 339, 936 10.1126/science.1230020. 23371555

[ref27] Awazu A. Segregation and phase inversion of strongly and weakly fluctuating brownian particle mixtures and a chain of such particle mixtures in spherical containers. Phys. Rev. E 2014, 90, 042308 10.1103/PhysRevE.90.042308. 25375495

[ref28] Ganai N.; Sengupta S.; Menon G. I. Chromosome positioning from activity-based segregation. Nucleic Acids Res. 2014, 42, 4145 10.1093/nar/gkt1417. 24459132 PMC3985638

[ref29] Tsalikis D. G.; Mavrantzas V. G. Size and diffusivity of polymer rings in linear polymer matrices: The key role of threading events. Macromolecules 2020, 53, 803 10.1021/acs.macromol.9b02099.

[ref30] Liu L.; Shi G.; Thirumalai D.; Hyeon C. Chain organization of human interphase chromosome determines the spatiotemporal dynamics of chromatin loci. PLOS Comput. Biol. 2018, 14, 1 10.1371/journal.pcbi.1006617. PMC6292649 30507936

[ref31] Tsalikis D. G.; Mavrantzas V. G.; Vlassopoulos D. Analysis of slow modes in ring polymers: Threading of rings controls long-time relaxation. ACS Macro Lett. 2016, 5, 755 10.1021/acsmacrolett.6b00259. 35614653

[ref32] Landuzzi F.; Nakamura T.; Michieletto D.; Sakaue T. Persistence homology of entangled rings. Phys. Rev. Research 2020, 2, 033529 10.1103/PhysRevResearch.2.033529.

[ref33] Michieletto D.; Sakaue T. Dynamical entanglement and cooperative dynamics in entangled solutions of ring and linear polymers. ACS Macro Lett. 2021, 10, 129 10.1021/acsmacrolett.0c00551. 35548984

[ref34] Sakaue T. Topological free volume and quasi-glassy dynamics in the melt of ring polymers. Soft Matter 2018, 14, 7507 10.1039/C8SM00968F. 30152832

[ref35] Mei B.; Dell Z. E.; Schweizer K. S. Microscopic theory of long-time center-of-mass self-diffusion and anomalous transport in ring polymer liquids. Macromolecules 2020, 53, 10431 10.1021/acs.macromol.0c01737.

[ref36] Shaban H. A.; Barth R.; Bystricky K. Formation of correlated chromatin domains at nanoscale dynamic resolution during transcription. Nucleic Acids Res. 2018, 46, e77 10.1093/nar/gky269. 29718294 PMC6061878

[ref37] Strickfaden H.; Zunhammer A.; van Koningsbruggen S.; Köhler D.; Cremer T. 4D chromatin dynamics in cycling cells: Theodor boveri’s hypotheses revisited. Nucleus 2010, 1, 284 10.4161/nucl.1.3.11969. 21327076 PMC3027035

[ref38] Agrawal A.; Ganai N.; Sengupta S.; Menon G. I. Chromatin as active matter. J. Stat. Mech.: Theory Exp. 2017, 2017, 014001 10.1088/1742-5468/aa5287.

[ref39] Saintillan D.; Shelley M. J.; Zidovska A. Extensile motor activity drives coherent motions in a model of interphase chromatin. Proc. Natl. Acad. Sci. U.S.A 2018, 115, 11442 10.1073/pnas.1807073115. 30348795 PMC6233076

[ref40] Nuebler J.; Fudenberg G.; Imakaev M.; Abdennur N.; Mirny L. A. Chromatin organization by an interplay of loop extrusion and compartmental segregation. Proc. Natl. Acad. Sci. U.S.A 2018, 115, E6697 10.1073/pnas.1717730115. 29967174 PMC6055145

[ref41] Liu K.; Patteson A. E.; Banigan E. J.; Schwarz J. M. Dynamic nuclear structure emerges from chromatin cross-links and motors. Phys. Rev. Lett. 2021, 126, 158101 10.1103/PhysRevLett.126.158101. 33929233

[ref42] Woodhouse F. G.; Goldstein R. E. Spontaneous circulation of confined active suspensions. Phys. Rev. Lett. 2012, 109, 168105 10.1103/PhysRevLett.109.168105. 23215137

[ref43] Put S.; Sakaue T.; Vanderzande C. Active dynamics and spatially coherent motion in chromosomes subject to enzymatic force dipoles. Phys. Rev. E 2019, 99, 032421 10.1103/PhysRevE.99.032421. 30999440

[ref44] Di Pierro M.; Potoyan D. A.; Wolynes P. G.; Onuchic J. N. Anomalous diffusion, spatial coherence, and viscoelasticity from the energy landscape of human chromosomes. Proc. Natl. Acad. Sci. U.S.A 2018, 115, 7753 10.1073/pnas.1806297115. 29987017 PMC6065008

[ref45] Shi G.; Liu L.; Hyeon C.; Thirumalai D. Interphase human chromosome exhibits out of equilibrium glassy dynamics. Nat. Commun. 2018, 9, 3161 10.1038/s41467-018-05606-6. 30089831 PMC6082855

[ref46] Fudenberg G.; Imakaev M.; Lu C.; Goloborodko A.; Abdennur N.; Mirny L. A. Formation of chromosomal domains by loop extrusion. Cell Rep 2016, 15, 2038 10.1016/j.celrep.2016.04.085. 27210764 PMC4889513

